# Feasibility of a school-based mental health program implementation to improve the status of depression and quality of life of mothers of children with autism spectrum disorders in urban Bangladesh: MENTHOL study

**DOI:** 10.1017/gmh.2022.16

**Published:** 2022-03-23

**Authors:** Aliya Naheed, Md. Saimul Islam, Meredith B. Brooks, Mary C. Smith Fawzi, Mir Nabila Ashraf, Helal Uddin Ahmed, M. M. Jalal Uddin, Kamrun Nahar Koly, Jerome T. Galea, Shaheen Akhter, Charles Nelson, Saima Wazed Hossain, Kerim M. Munir

**Affiliations:** 1 Initiative for Non-communicable Diseases, Health System and Population Studies Division, icddr,b 1212, Dhaka, Bangladesh; 2 Department of Global Health and Social Medicine, Harvard Medical School, Boston, USA; 3 National Institute of Mental Health, Dhaka, Bangladesh; 4 National Institute of Neurosciences & Hospital, Dhaka, Bangladesh; 5 School of Social Work, University of South Florida, FL, USA; 6 Institute of Paediatric Neurodisorder and Autism (IPNA), Bangabandhu Sheikh Mujib Medical University (BSMMU), Dhaka 1000, Bangladesh; 7 Division of Developmental Medicine, Boston Children's Hospital, Boston, USA; 8 Department of Pediatrics, Harvard Medical School, Boston, MA, USA; 9 Shuchona Foundation, Dhaka 1205, Bangladesh; 10 Adrian Dominican School of Education, Barry University, Miami, USA; 11 Department of Psychiatry, Harvard Medical School, Boston, MA 02115, USA

**Keywords:** Bangladesh, children with autism, depression, mothers, psychological counseling

## Abstract

**Background:**

We assessed the feasibility of implementing psychological counseling services (PCS) for mothers of children with autism spectrum disorders (ASD) integrated within special education settings in urban Bangladesh.

**Method:**

In two special education schools for ASD in Dhaka City, trained female psychologists screened mothers using the Patient Health Questionnaire (PHQ-9). PCS was administered to all the mothers irrespective of a diagnosis of depression. Mothers with a PHQ-9 score >4 who met criteria for a major depressive episode (MDE) based on the DSM-IV Structured Interview Axis I Disorders (SCID-I) were also administered skill-building training through monthly home visits to support ASD care. The level of depression was assessed by the Depression Measurement Scale (DMS), and quality of life (QoL) was measured by Visual Analogue Scale (VAS) of EQ5D5L scale before and after PCS.

**Result:**

Among 188 mothers enrolled in the study, 81 (43%) received PCS, and 27.1% (22) had MDE. In the first month, 73 sessions were scheduled and 60 completed (85%). In the last month, 53 sessions were scheduled and 52 completed (98%). The mean DMS score decreased from 79.5 ± 23 to 60 ± 20 (*p* = 0.004), and DMS scores were significantly higher among mothers with MDE (97.8 ± 12.1 *v.* 69.9 ± 22.1; *p* < 0.001) compared to those without MDE (72.7 ± 22.6 *v.* 56.1 ± 18.1; *p* = 0.003). The mean VAS score improved from 70.3 ± 14.1 to 80.2 ± 13.3 (*p* = 0.001) between the first and the last session. Changes in DMS were negatively correlated with changes in VAS scores (*β:* −0.213, 95% CI 0.370 to −0.056).

**Conclusion:**

Within special education schools for ASD in urban Bangladesh, it was feasible to administer an integrated program of PCS for mothers of children with ASD by trained psychologists who were able to screen and intervene to reduce their level of depression and improve their quality of life.

## Introduction

The global burden of depression in 2019 accounted for 5.5% of the disability-adjusted life years (DALYs), with 110 million DALYs in the South Asia region alone, women were affected more than men (IHME, 2021). Among the 322 million people affected worldwide (Friedrich, 2017), according to the World Health Organization (WHO), 86 million live in the South-East Asia region, and 75% of people with depression living in low- and middle-income countries (LMICs) do not receive any treatment (WHO, 2017; Hariyanto and Kurniawan, 2020).

In LMICs, the prevalence of depression among mothers of children with neurodevelopmental disorders ranges from 50% to 75% (Zhou *et al*., 2019; Naheed *et al*., 2020; Bourke-Taylor *et al*., 2021*a*). For the neurodevelopmental group overall, the global burden of depression is highest among mothers of children with autism spectrum disorders (ASD) (Weitlauf *et al*., 2014). A recent study from Bangladesh demonstrated that about 50% of mothers of children with ASD experienced major depressive disorder (MDD), and half of the mothers with MDD presented with a concurrent major depressive episode (MDE) (Naheed *et al*., 2020).

According to the Center for Disease Control and Prevention (CDC), one in 54 children in north america have ASD (Rockville, MD, March, 2020). A systematic review in South Asia reported an ASD prevalence range of 0.09–1.07% (Hossain *et al*., 2017; WHO, 2021). In Bangladesh, two national surveys have highlighted the burden of ASD at the community level (Hossain *et al*., 2017; Akhter *et al*., 2018; Kamruzzaman *et al*., 2019); in 2013, the prevalence of ASD was 15.5 per 10 000 children, with higher level in urban (300 per 10 000 children) than rural (6.8 per 10 000 children) areas (NCDC *et al*., 2013); by 2017, the prevalence of ASD in a rural community increased to 7.5 per 10 000 (Akhter *et al*., 2018).

Research shows that parenting a child with ASD is negatively associated with parental quality of life (QoL) (Dardas and Ahmad, 2014; Kuhlthau *et al*., 2014). Furthermore, a high burden of depression is more likely to negatively impact the ability of parents to care for children at home (Singh *et al*., 2017; Gobrial, 2018). Since mothers are often the primary caregivers of children with ASD, and depression and low QoL are both highly prevalent among them (Naheed *et al*., 2020), there is an urgent need for provision of mental health interventions within ASD care systems (Karr *et al*., 2017; Althoff *et al*., 2019). With the onset of COVID-19 pandemic, parents of children with ASD have faced an increased mental health crisis (Baghdadli *et al*., 2021), with disproportionate impact in LMICs where availability of mental health care supports is severely limited (Oomen *et al*., 2021). There is an important need for identifying a feasible implementation strategy that can also be sustained during public health emergencies, to address the mental health needs of mothers of children with ASD living in resource-poor settings.

Symptoms of maternal depression characteristically range from a sense of hopelessness, withdrawal, to fluctuations in mood, loss of focus, and unexplained aches and weariness (Slomian *et al*., 2019). A meta-analysis found that psychoeducational interventions are beneficial in improving the mental health of mothers (Bourke-Taylor *et al*., 2021*b*) including those with depression encountered in primary care settings (Linde *et al*., 2015). The WHO recommends advanced psychosocial interventions to manage mild to moderate depression as the first line of treatment, including skills training offered for parents of children with neurodevelopmental disorders (WHO, 2016). As in many other South Asian LMICs, mental health is not a priority in Bangladesh in routine primary care. Mental health services are generally offered in tertiary teaching hospitals as well as the National Institute of Mental Health Institute (NIMH, 2020). There are currently no dedicated psychological counseling services (PCS) in the country for mothers of children with ASD. Furthermore, many mothers avoid receiving care at mental health facilities due to fear of stigmatization together with the lack of support available in the hospital environment for their accompanying children (Naheed *et al*., 2020).

We conducted a feasibility study integrating PCS for mothers in two special education schools offering ASD care. Additionally, we assessed changes in mental health status and QoL among mothers following the administration of PCS. The research questions included (1) Is an implementation of a PCS program feasible targeting the mental health needs of mothers of children with ASD in a special education setting in urban Bangladesh?; and (2) Will a PCS program improve the mental health status and QoL of mothers of children with ASD?

## Methods

A pilot feasibility study with a pre-post intervention design was implemented. The detailed methodology has been described previously (Naheed *et al*., 2017). Briefly, the study was conducted in two specialized education schools in Dhaka City that have provided services for children with ASD for over a decade. Mothers who participated in the study were at least 18 years of age, had a child with ASD enrolled in the schools, and provided voluntary informed consent. Trained research staff conducted surveys among mothers before (baseline) and after the intervention to assess their level of depression. The nine-item Patient Health Questionnaire (PHQ-9) was used to screen for depression. Mothers with PHQ-9 scores >4 were further assessed for confirmatory diagnoses of MDE using the Structured Clinical Interview Axis-I Disorders (SCID-I), Non-Patient (SCID-I NP), the Diagnostic and Statistical Manual of Mental Disorder, 4th edition (DSM-IV) research version (Kroenke *et al*., 2001; Kupfer *et al*., 2008). Culturally validated EQ-5D-5L™ tools were applied irrespective of the presence of MDE and provided measures of five dimensions of QoL, including mobility, self-care, usual activities, pain/discomfort, and anxiety/depression; each dimension was assessed on three levels (no problems, some problems, or extreme problems). EQ-5D also included a Visual Analogue Scale (VAS) that allowed self-rating on a 20 cm vertical scale with endpoints of ‘best imaginable health state’, set at 100, and ‘worst imaginable health state’ set at 0.

### Intervention

A trained female psychologist offered face-to-face PCS (intervention) during school hours over a 6-month period. This was done at a designated space for any mother who voluntarily agreed to participate, regardless of history of depression or any other mental health condition. The content of the psychological management module consisted of psychoeducation, assessment of the mothers' strengths and weaknesses, sharing the management plan, cognitive restructuring, behavior therapy-graded activity, developing skills to engage mothers in community-related activities, group meditation, and mental health awareness sessions. The psychologists organized workshops in each school every 2 months, and group meditation sessions were held after every workshop.

The subgroup of mothers with an MDE additionally received skills-building training services by a special education counselor who paid monthly home visits to support the care of their ASD child at home (Naheed *et al*., 2017). Thus, there were two subgroups: mothers with MDE who received both the PCS and home-based training, and mothers without MDE who only received the PCS intervention. A psychiatrist visited each school monthly and reviewed the records maintained by the PCS psychologists regarding the need for any additional care of mothers identified with MDE. Mothers identified with MDE with any safety concern (e.g. suicidal ideation, suicidal intent or plan, or a prior history of suicide attempt at baseline assessment) were referred to the National Institute of Mental Health and Hospital, Bangladesh (NIMH,B) for specialized mental health care as per the study protocol and the standard of care practice (Nuri *et al*., 2018).

Each psychologist assessed a mother's depression by applying a locally customized Beck Depression Inventory (BDI) tool, known as Depression Measurement Scale (DMS), routinely used by psychologists in Bangladesh (Uddin and Rahman, 2005). DMS measures the level of depression on a scale between 30 and 150 (DMS score) to categorize the severity of depression into minimal (30–100), mild (101–114), moderate (115–123), and severe (124–150) at the beginning of the first session (pre-intervention), and again after the final session (post-intervention) (Uddin and Rahman, 2005).

Psychologists maintained an appointment book to register the mothers who requested the service and scheduled PCS sessions according to the convenience of the mothers. When the DMS score of a mother reduced to minimal, that mother was considered to have a healthy mental health state and was not invited to attend further PCS sessions. An exit interview was conducted after the last session to understand the mothers' perspectives about acceptability, barriers, benefits, affordability, and demand of integrating PCS in schools. Online Supplementary Fig. S3 provides the template for the intervention description and replication (TIDieR) checklist, which explains the details of the intervention.

### Data analysis

Categorical variables were reported by frequency distribution, and continuous variables were reported by summary statistics, such as mean, median, standard deviation (SD), and range according to the nature of the data. The χ^2^ test was applied for bivariate analysis of maternal factors to estimate the difference between the mothers receiving and those not receiving the intervention. Linear regression was performed to identify the associations of changes in depression level (Post DMS-Pre DMS) with the factors that were significantly different between the mothers who did or did not receive the intervention. The EQ-5D index was calculated from the five domains of EQ-5D-5L, and the UK-based preferred weight was applied, as the country-specific population-based weight of EQ-5D index is not available for Bangladesh. The UK algorithm was calculated using a hybrid model of data acquired via a time-trade-off and discrete choice experiment. The algorithm's possible values varied from −0.281 to 1, with values <0 representing conditions regarded to be worse than death. The Wilcoxon rank-sum test was used to measure the mean difference of DMS and QoL among the intervention mothers from pre- to post-PCS sessions. An *α* level of <0.05 was considered statistically significant for all tests. All statistical analyses were done using the SPSS Version 21.0. Less than 1% of data were missing, and missing data were excluded from the analysis of those variables.

### Ethics

Ethical clearance for this study protocol was obtained from the Ethical Review Committee of icddr,b (International Centre for Diarrhoeal Disease Research, Bangladesh) and was exempted from further human subject research review following review by the Director of Compliance of the IRB at the Boston Children's Hospital. All study participants were enrolled in the baseline survey, end line survey, and the intervention following written informed consent.

## Results

### Socio-demographic characteristics of the study participants

The study was conducted between January 2017 and December 2018. We obtained a list of 310 children with ASD enrolled in two schools, and 198 of their mothers responded to our invitation to participate (63.9%). Among the responders, 188 mothers participated in the survey (94.9%) and 10 mothers declined to participate (5.1%). The average age of the mothers who responded to the survey was 41 (±7.5) years; 58 mothers had a single child (31%), and 130 mothers had more than one child (69%).

The majority (85%) of the mothers lived in a nuclear family with an average size of 4 (±1.3) family members, and 15% of mothers lived in a joint family with extended family members at an average size of 7 (±3) members. Most of the mothers (96%) completed high school education, and the majority of them were homemakers (71%). There was a single wage earner in 130 families (69%), and the mother was the single wage earner in 11 families (8.5%). The median monthly income of a family was US$956 (range = US$36–41 836); 14 (7%) families reported to have an income below the poverty level as defined by the World Bank (i.e. less than US$1.90 per day per capita) (World Bank, 2011) (Table 1).

**Table 1.** Socio-demographic characteristics of the participants at the baseline survey (*N* = 188)

Characteristics of mothers	Total (*N* = 188)	PCS service received/intervention mothers (*n* = 81)	PCS service not received/non-intervention mothers (*n* = 107)
Age, mean ± SD	41 ± 7.5	40 ± 8.0	42 ± 7.0
Education, *n* (%)			
Did not complete primary education	3 (1.6)	1 (1.2)	2 (1.8)
Completed primary education (grade 5–10)	6 (3.2)	2 (2.4)	5 (4.5)
Completed 10 years of schooling (grade 11–12)	32 (17.0)	14 (17.2)	18 (16.2)
Completed a college degree	44 (23.4)	18 (22.2)	29 (26.1)
Completed a post-graduation degree	103 (54.8)	46 (56.7)	57 (51.4)
Occupation, *n* (%)			
Homemaker	134 (71.3)	62 (76.5)	75 (67.6)
Service holder	46 (24.5)	17 (21.0)	30 (27)
Business	8 (4.3)	2 (2.5)	6 (5.4)
Previously involved with any service if not in service, *n* = 117 (%)	46 (34.3)	22 (27.1)	24 (32)
Marital status, *n* (%)			
Married	175 (93.0)	75 (92.6)	103 (92.8)
Single mothers (widow/divorced/separated)	13 (7.0)	6 (7.4)	8 (7.2)
Spouse age^$^ (mean ± SD)	47.3 ± 8	46.0 ± 7.0	49.0 ± 8.0
Spouse education^$^, *n* = 175 (%)			
No education/below primary education	4 (2.3)	0(0)	4 (3.8)
Completed primary education (class 5–10)	4 (2.3)	2 (2.7)	3 (2.9)
Completed 10 years of schooling (class 11–12)	12 (6.9)	9 (12.3)	3 (2.9)
Completed a college degree	42 (24.3)	18 (24.7)	25 (23.8)
Completed a post-graduation degree	114 (64.2)	44 (60.3)	70 (66.7)
Spouse occupation^$^ (*N* = 175)			
Services	130 (75.1)	56 (76.7)	78 (74.3)
Business	38 (22.0)	15 (20.5)	24 (22.9)
Retired/currently not earning	5 (2.9)	2 (2.7)	3 (2.9)
Number of wage earners in the family, *n* (%)			
1	130 (69.1)	63 (77.8)	71 (64)
2	53 (28.2)	16 (20.8)	37 (33.3)
3	5 (2.7)	3 (3.7)	3 (2.7)
Main wage earner in the family, *n* (%)			
Participant herself	16 (8.5)	10 (12.3)	8 (7.2)
Husband	166 (88.3)	69 (85.1)	99 (89.2)
Other members	6 (3.2)	2 (2.4)	4 (3.6)
Gross monthly income of the family (US$^a^), median (IQR: 25th–75th quartile)^b^	956 (36–41 836)	878.8 (597.8–1644.0)	1004.2 (478.2–1793.4)

a 1 US$ = 83.75 Bangladeshi Taka (BDT) in 2018 according to US$ to BDT spot exchange rate.

b Information from four participants was missing.

$ The biological father of the child.

### Types of support available to mothers for the care of a child with ASD

Nearly 66% of the mothers (*n* = 124) mentioned that they had someone at home to help them take care of their children with ASD, including their husbands (44%), other children (32%), extended family members (14.5%), housekeepers (41.9%), and teachers (0.81%). About 47% of the mothers reported problems while taking care of their children at home, including being too occupied with her child to care for herself (57%), aggressive behaviors in the child requiring close monitoring (54.4%), and the child having limited self-care skills (10.1%) causing further challenges at home. Nearly half (48.9%) of the mothers also reported experiencing negative attitudes toward their child when taken outside the home, and most (82%) reported avoiding social events (online Supplementary Table S1). Over half (57.4%) of the mothers reported difficulties managing their child when taken outside of the home. About two-thirds (64.9%) of mothers reported having received specialized training on how to take care of their child with ASD, but the difficulties faced by these mothers were no different from those who did not receive the training (47.5% *v.* 44.5%; *p* = 0.689).

### Health care seeking of mothers for children and self

Nearly half (49%) of the mothers reported their child being sick in the past 6 months and having to go to various providers to seek medical care; with 57% seeking care from a doctor in a private office, 32% from a private hospital, 23% from a government hospital, 19% from a clinic, and 2% from a pharmacy. The majority of them (75%) reported satisfaction with the services received for their child.

Fifty-three percent of mothers reported experiencing a physical illness over the past 6 months. The majority (66%) of the mothers reported various unspecified health conditions such as physical weakness, skin problems, hormonal problems, urinary tract infections, and fever, and 34% reported a specific condition including elevated blood pressure, diabetes, thyroid problems, chronic kidney disease, and heart disease. Among those who reported physical illnesses, 78.4% had received treatment, with 52% in a private office, 32% at a private hospital, 14% at a public hospital, 14% at a general clinic, and 4% at a pharmacy outlet. The majority of them (75%) were satisfied with their providers (online Supplementary Table S1).

When asked specifically about mental health concerns, 53% of mothers reported having an experience that caused mental distress in the last 6 months. Sixty-two percent of mothers who had a physical illness also reported a mental health issue. Among those who reported a mental health issue, about half (47.9%) reported not doing anything about it; 21% reported adopting some means to reduce their mental distress, e.g. reading a book, watching television, increasing social interaction, listening to music, prayer, and meditation; 18.8% reported sharing their concerns with others; 17.7% sought the advice of a doctor or a psychiatrist; and 3% reported distancing themselves from social situations (online Supplementary Table S1).

### Intervention fidelity

**Fig. 1.**
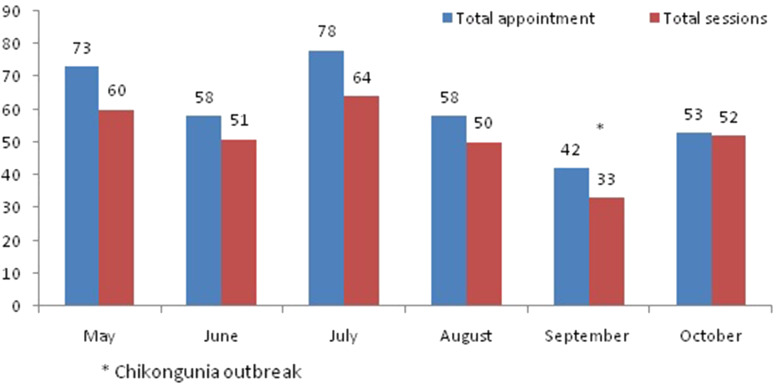
Response of the mothers to the intervention offered at the schools by month.

Between May and October 2018, 81 mothers received at least one PCS session from the counseling program at the schools during the intervention period. In the first month of the intervention period, 73 sessions were scheduled, and 60 (85%) completed. In the last month of the intervention, 53 sessions were scheduled, and 52 (98%) completed. The number of sessions increased over time, except during a Chikungunya outbreak in September 2017, when 42 sessions were scheduled and only 33 (78%) sessions could be conducted (Fig. 1).

Altogether, 318 PCS were conducted in two schools in 24 weeks; and on average, a counselor conducted 2.2 sessions per school per day. In addition, the psychologists held 14 workshops (group sessions) with the number of attendees varying from 14 to 45 participants per session. Sixteen mothers were provided treatment by psychiatrists during their consultation visits at the schools, four of them received pharmacological treatment, and one of them obtained treatment from NIMH,B. Overall, 60 (75%) mothers participated in the exit interviews, and the majority (95%) of them were satisfied with the intervention. Ninety percent of mothers reported that the PCS met their expectations in terms of improvement of their mental health and experiencing self-empowerment. They also reported improvement in their daily life after attending PCS (88.3%), including experiencing increased self-confidence (81%), self-care (79%), and taking an interest in daily routines (77%). Three-quarters of mothers reported having done something new that offered mental peace. Most (86.7%) of the mothers reported that the PCS helped them take better care of their children with ASD, and that it helped remove negative thoughts about their child (online Supplementary Tables S2 and S6).

Although the services were offered free of cost, 31 mothers (38%) reported additional costs: 21 mothers, for further spending related to attending PCS sessions; six mothers, for facilitating social interaction of the child; and four mothers, for medical care linked to the PCS. On average, the mothers spent nearly US$1.00 per session, and most (97%) of the mothers supported continuation of the PCS at the schools. When asked about their opinion for further improvement of the PCS, 73.3% of mothers recommended additional measures, such as arranging an after-school program, offering therapy for their children, involving other family members, and further strengthening training opportunities. Most of the mothers (86.7%) did not report facing any challenges in attending the PCS sessions, except for a small group (21.7%) who reported lack of family support or lack of time due to other priorities (15.0%) or hesitation to seek care due to social stigma about ASD (11.7%) as barriers (online Supplementary Table S3).

### Number of PCS sessions offered to the intervention mothers

Among 81 intervention mothers, 50% attended a minimum of four PCS sessions (range 1–13). After every PCS session, the psychologist conducted a DSM assessment, where a mother's participation was discontinued if she demonstrated an improvement in mental health status to the minimal level. As such, five of the 81 mothers (6%) showed improved mental health status after the first session, and were excluded from further sessions. Among the remaining 76 mothers who were invited to attend the second session, 31 mothers were excluded from additional sessions due to improvement, and 45 mothers were invited to attend the third session. After the fourth session, 54 (67%) of mothers had improvement in their mental health state, and after the eighth session, 80 (98.7%) of the mothers showed improvement. One mother who did not improve after attending her eighth session was offered a total of 13 sessions until the end of the intervention period and her depression status did not improve (Table 2).

**Table 2.** DMS score changes among the intervention mothers according to the PCS sessions received

# sessions	Number of mothers attended the session (*N*)	Number of mothers whose mental health state improved after PCS (*N*)	DMS score at first session mean ± s.d.	DMS score at the last session mean ± s.d.	Mean difference (first *v.* last)	Incremental DMS score per incremental session	*p* value^a^
1st session	81	5	80 ± 23	63 ± 21	17		*p* < 0.001
2nd session	76	31	80 ± 23	61 ± 20	19	2	*p* < 0.001
3rd session	45	11	86 ± 21	65 ± 21	21	2	*p* < 0.001
4th session	34	7	90 ± 21	67 ± 21	23	2	*p* < 0.001
5th session	27	4	90 ± 22	66 ± 22	24	1	*p* < 0.001
6th session	23	8	94 ± 21	67 ± 23	27	3	*p* < 0.001
7th session	15	3	100 ± 11	71 ± 24	29	2	*p* < 0.001
8th session	12	11	99 ± 10	71 ± 14	28	1	*p* < 0.001
9th session	1	1	94	54	40		

^a^Paired sample *t* test for mean difference; statistical significance at *p* < 0.05.

### Change in depression status among intervention mothers

**Fig. 2.**
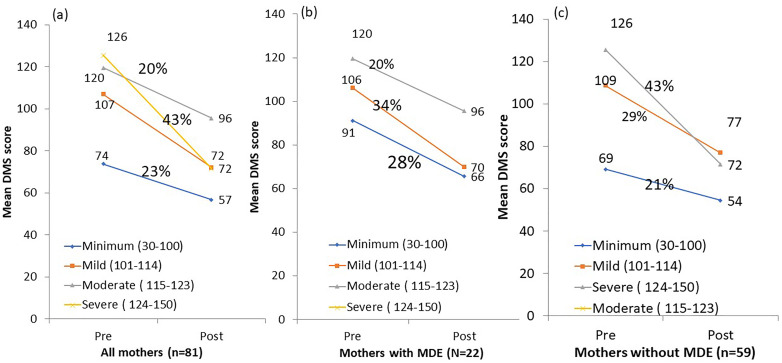
Mean change of DMS score by severity of depression among mother (*a*) overall, (*b*) mothers with major depressive episode, and (*c*) mothers without major depressive episode.

The overall proportion of MDE among the intervention mothers reduced from 17.2% to 6.2% (*p* = 0.029) following the 6-month intervention period. The average DMS score among 81 mothers had decreased significantly between the first and the last PCS sessions (79.5 ± 23.1 *v.* 60.0 ± 20.1; *p* = 0.004). Among 22 mothers diagnosed with MDE, the mean DMS score reduced from 97.8 (±12.1) at the first session to 69.9 (±22.1) at the last session, with a 40% decrease. Among 59 mothers who did not have an MDE, the mean DMS score reduced from 72.7 (±22.6) at the first session to 56.1 (±18.1) at the last session, leading to a 23% decrease from baseline DMS score (*p* < 0.001). The pre-post difference of DMS scores among the mothers with MDE was 20% higher than the pre-post differences of DMS score among the mothers without MDE (online Supplementary Table S4). Mothers with MDE received more sessions than those without MDE (7.0 ± 2.0 *v.* 3.0 ± 2.8; *p* < 0.001). The level of DMS score reduced from a mild, moderate, or severe depression level to a minimum level among 27.4% of mothers who had MDE compared to 6.8% of mothers who did not have MDE (Fig. 2).

### Change in QoL among intervention mothers

**Fig. 3.**
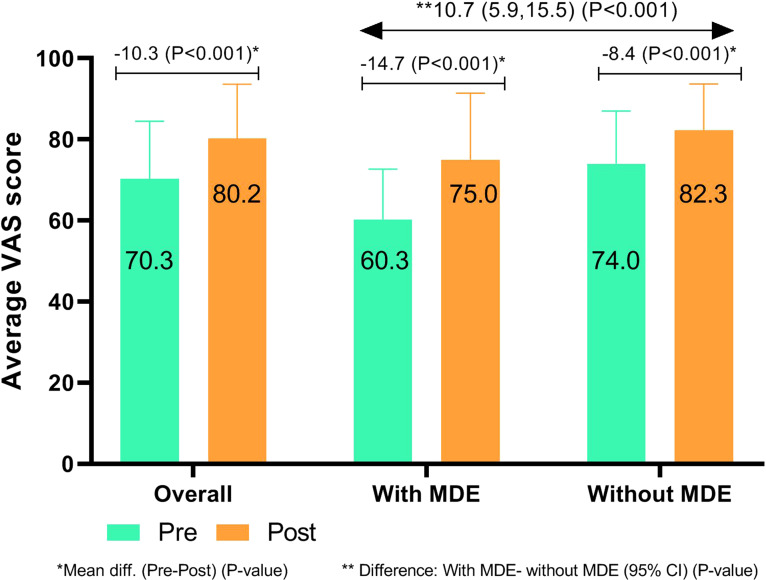
Mean change of quality of life (EQ5D VAS scale) among mother who received psychosocial service, (*a*) overall, (*b*) mothers with major depressive episode, and (*c*) mothers without changes in quality of life before and after the intervention among the mothers.

The average EQ-5D index for QoL among the intervention mothers was 0.78 (±0.2) at baseline and 0.84 (±0.15) at end line (*p* < 0.001). Statistically significant improvement was observed for ‘pain/discomfort’, which reduced from 57.4% to 50.9% (*p* < 0.001), and for ‘anxiety/depression’, which reduced from 50.0% to 37.3% (*p* < 0.001). However, the proportional changes were not observed in case of mobility, self-care, and usual activities from baseline to end line. QoL was higher among intervention mothers who had reported not facing difficulties with childcare either at or outside the home compared to the intervention mothers who reported facing such difficulties. The mean (±s.d.) VAS score improved from 70.3 (±14.1) to 80.2 (±13.3) after intervention (*p* = 0.001) (Table 3). The mean difference of VAS score from pre- to post-intervention was higher among mothers with MDE when compared to mothers without MDE (mean difference: 10.7; 95% CI 5.9–15.5; *p* < 0.001) (Fig. 3). The mean VAS score was negatively correlated with changes in DMS score from pre- to post-counseling PCS sessions (*β* coefficient: −0.213, 95% CI −0.370 to −0.056; *p* = 0.031), suggesting improvement in QoL among the intervention mothers along with a reduction in depression (online Supplementary Table S5).

**Table 3.** Changes in quality of life before and after the intervention among the mothers

Characteristics	Before intervention (*N* = 81; %)	After intervention (*N* = 81; %)	*p* value^a^
Quality of life (EQ5D3L)			
Mobility			
No problem in walking	45 (75.0)	48 (81.4)	0.185
Slight problem with walking	10 (16.7)	10 (16.9)	
Has a problem walking	5 (8.3)	1 (1.7)	
Self-care			
No problem in self-care	54 (90.0)	55 (93.2)	0.608
Slight problem in wearing cloth or washing	5 (8.3)	3 (5.1)	
Have problem in wearing cloth or washing	1 (1.7)	1 (1.7)	
Usual activities			
No problem in usual activities	49 (81.7)	51 (86.4)	0.051
Slight problem in usual activities	10 (16.7)	7 (11.9)	
Have problem in usual activities	1 (1.7)	1 (1.7)	
Pain/discomfort			
No pain or discomfort	26 (43.3)	29 (49.2)	0.016^a^
Slight pain or discomfort	31 (51.7)	29 (49.2)	
Have problem pain or discomfort	3 (5.0)	1 (1.7)	
Anxiety/depression			
No anxiety/depression	30 (50)	37 (62.7)	*p* < 0.001
Slight anxiety/depression	25 (41.7)	21 (35.6)	
Have problem anxiety/depression	5 (8.3)	1 (1.7)	
EQ5D index, mean (SD)	0.78 (0.20)	0.84 (0.15)	*p* < 0.001^b^

^a^McNemar–Bowker χ^2^ test applied for the paired proportion test.

^b^Paired sample *t* test applied for the mean difference; statistical significance at *p* < 0.05; *p* < 0.001.

## Discussion

To our knowledge, this is the first study in an LMIC setting that has implemented school-based mental health services for mothers of children with ASD. The study demonstrates that PCS is a preliminarily effective means for managing depression among a highly vulnerable group of mothers of children with ASD. The levels of depression were significantly reduced among both sets of mothers with and without MDE. The demand for counseling sessions was high, and nearly half the mothers received four or more counseling sessions. The QoL improved across all the five dimensions of EQ-5D and the mothers' self-rated health status as measured by VAS. At baseline, one out of three mothers had depressive symptoms screened by PHQ-9, and a favorable effect was observed with improved mental health across all mothers who had received the service, irrespective of having clinical depression. Overall, the study demonstrated that integrating PCS in schools offering ASD services was acceptable, affordable, with a demand for such services among the mothers.

The PCS entailed an effective intervention for lowering maternal depression and enhancing parenting skills simultaneously (Goodman and Garber, 2017). A meta-analysis found that psychoeducational programs can improve health-related self-efficacy, positive health behavior, coping style, and life satisfaction among mothers of children with ASD (Bourke-Taylor *et al*., 2021*a*). Evidence suggests that parent-mediated programs and parental psychoeducation are helpful for increasing parent–child interactions, improving aspects of maternal awareness of ASD and related symptoms, and developing support for enhancing children's daily living activities (Shire *et al*., 2016; Koly *et al*., 2021).

A systematic review on PCS globally suggests that interventions lasting for more than 3 months exhibit more positive effects compared to shorter duration interventions (Forsman *et al*., 2011). Evidence also suggests that combined prevention and promotion of mental health within the overall public health system can reduce the stigma for receiving the service and can provide positive outcomes (World Health Organization, 2002; Safarabadi-Farahani *et al*., 2016). The mothers who participated in our intervention received counseling sessions over a 6-month period according to their needs; the severity of depression had reduced to 20% from pre- to post-intervention counseling session, and the PCS had demonstrated a better effect among mothers with a more recent MDE event.

The findings also demonstrate that most mothers received treatment from providers for physical care, but felt mental distress due to negative events in the past 6 months. The average depression score significantly changed among mothers who had comorbidities including hypertension and diabetes, suggesting that PCS may play a significant role for these mothers by providing support to cope with both physical and mental illnesses. The findings corroborate evidence from other studies demonstrating that concurrent management of physical and mental health in long-term conditions is important, and psychological interventions can provide benefits for both physical as well as mental health for the parents of children with special needs (Gupta and Singhal, 2005; Catalano *et al*., 2018).

A randomized trial suggested that parent education, counseling and skills-training programs for parents of young children newly diagnosed with ASD significantly improve parental mental health (Tonge *et al*., 2006). In our study, the majority of mothers received training on child care as part of the routine program offered at schools. Still, only a few reported participating in a program that provides mental health care. We found that the average DMS score of depression had significantly reduced among those mothers who received training and service related to child care. One possible explanation of these findings is that the training program and the PCS received by mothers had played a significant role as an early intervention in helping them to manage their depression. The children of mothers with MDE may have had a better performance than children of mothers without MDE. This may not be surprising because mothers who had MDE received additional home-based training, which may explain the improvement of behavioral management of their children. Evidence supports that a parent-mediated intervention program is effective for improving the performance of children with neurodevelopmental concerns (Koly *et al*., 2021).

Another study conducted in Bangladesh reported that the QoL of mothers of children with ASD was generally poor and that their higher depression level was correlated with lower QoL (Naheed *et al*., 2020). This study demonstrated that the mother's QoL improved as depression was reduced and that there was a dose–response relationship between depression status and the number of counseling sessions received, rendering better improvement in QoL among mothers with MDE than those without. One explanation for the findings could be that the demand for counseling was higher among the mothers who had clinical depression, and their ease of access to mental health services – augmented by monthly home visits – enabled these mothers to better manage their child at home, also resulting in an improved overall QoL. Converging evidence suggests that PCS intervention is an effective early strategy to improve the QoL of mothers as primary caregivers of their children (Hennessy *et al*., 1994; Safarabadi-Farahani *et al*., 2016) and our study supports that PCS could be feasible in LMICs. A systematic review on parental QoL reported that the support system could improve the QoL of mothers of children with ASD (Vasilopoulou and Nisbet, 2016). Thus, integrating skills-building programs for ASD care for mothers within the PCS service would be a sensible strategy to address maternal mental health and improve the overall QoL of those mothers at risk for depression, reporting physical limitations and not yet diagnosable anxieties and early symptoms of depression.

The study needs to be viewed in the context of several limitations. First, this is not a randomized controlled trial that tested a specific hypothesis, but the study aimed to assess program implementation feasibility. The findings on improvement in mental health status and QoL are based on pre- and post-intervention changes in the context of two special education schools. Second, the results are specific to a setting where the mothers were relatively young, highly educated, and lived in high-income families in Dhaka City, and is not representative of the entire population of mothers of children with ASD in urban Bangladesh. Therefore, the interpretations cannot be applied to mothers of children with ASD in less affluent circumstances in Dhaka or elsewhere. Nonetheless, the high proportion of mothers with MDE suggests that the need for such programs in more resource-poor or rural contexts is likely to be even greater. Third, about 50% of the mothers who had their children with ASD enrolled in the two schools did not participate in the program despite the relative ease of access to the service, potentially related to stigma or other social reasons that could not be explored. This is a finding observed in prior research and may entail the need for a more assertive outreach for young mothers caring for children with ASD (Oner and Munir, 2020). We could not verify if the effect of PCS on depression observed among the intervention mothers would be similar among the non-intervention mothers who did not avail the PCS service despite being offered at the schools. However, we have documented that only a few among the non-intervention mothers had an MDE, which indicates that this strategy has potentially removed the barriers attached to the stigma about their seeking mental health support and other influences may be at play. Lastly, various factors such as physical health, environmental, financial, social, cultural, and family barriers may contribute to mental health issues among mothers of children with ASD that require risk mitigation, which was beyond the scope of the current program.

The study also has several strengths. First, intervening for depression among mothers of children with ASD following structured assessments, including the use of DSM-IV SCID-I questionnaires, is unique in an LMIC context. Second, the implementation of the PCS was integrated within a naturalistic special education ASD care setting. Third, the program could be efficiently delivered to mothers of children with ASD through voluntary participation. Integrating a very simple intervention that included offering routine PCS in school settings and monthly follow-up of mothers with MDE at home also demonstrated its positive impact on QoL, as well as mental health. Fourth, the positive findings of the program's implementation can be extended to other LMICs and low-resource settings as part of a global strategy (Islam and Biswas, 2015; Munir *et al*., 2016). Finally, the feasibility assessment of the PCS intervention is particularly salient in rural settings in Bangladesh and other LMICs.

In this study, the intervention mothers reported improvements in their children in terms of their exhibited behavior, communication, and vocational skills, as well as reduced dependence for daily living activities. Although not statistically significant, the direction of the results indicates collateral benefits of the PCS as a potential intervention to support both the child with ASD and their mothers at home, which would be crucial during emergencies that lead to constrained circumstances at home (WHO, 2020; Baghdadli *et al*., 2021). Indeed, the closure of schools due to COVID-19 had attributed to major psychological and behavioral problems among children and adolescents with ASD in South Asia (Omar *et al*., 2017; Zarrin, 2020; Baweja *et al*., 2021; Rohanachandra, 2021; Sharma *et al*., 2021). Thus, digital customization of the PCS at ASD school settings and further research could demonstrate a pragmatic solution for providing mental health care to mothers of children with ASD, which can be a sustainable solution during situations of health resource constraints such as the current pandemic, as well as an effective way to reach mothers in rural and remote areas during typical situations.

## Conclusion

The PCS intervention was feasible and acceptable among the mothers of children with ASD in an urban setting of Bangladesh. The severity level of depression was reduced among the mothers with and without major depression disorders, and significant improvement in overall QoL was observed. There is a pressing need for larger-scale research for broader implementation and targeting of depression in mothers of children with ASD, and to expand the availability of empirically-supported, cost-effective, non-pharmacological treatments in the care of parents of children with ASD.

## Data Availability

Data sharing will be handled upon request according to the data sharing policy of icddr,b.

## Supplementary material

For supplementary material accompanying this paper visit https://doi.org/10.1017/gmh.2022.16. click here to view supplementary material
