# Supplementary material for: Feasibility of a school-based mental health program implementation to improve the status of depression and quality of life of mothers of children with autism spectrum disorders in urban Bangladesh: MENTHOL study
Source: Glob Ment Health (Camb). 2022 Mar 23;9:146–56. doi: 10.1017/gmh.2022.16 (PMC9806996; doi:10.1017/gmh.2022.16)
Supplement: Supplementary file 1 [file S2054425122000164sup001.docx]

**Supplementary files**

**Supplementary Table 1.** Types of support available to the mothers for taking care of a child at home and outside of home

| Characteristic | Total n=188 | Percentage (%) | Intervention mothers  n=81 | Percentage (%) |
| --- | --- | --- | --- | --- |
| *Support for mother from family* |  |  |  |  |
| Average family member at household, mean(SD) | 5 (2) |  | 4.6 (1.7) |  |
| Types of family |  |  |  |  |
| Nuclear family | 102 | 63.7 | 67 | 82.7 |
| Joint family | 28 | 14.9 | 14 | 17.3 |
| Someone takes care of the child at home when mother needs go out child^$^ | 124 | 66.0 | 73 | 65.8 |
| Type of family members support mothers with childcare at home, *n=124(%)*  *(multiple response) ^$^* |  |  |  |  |
| Spouse | 55 | 44.4 | 34 | 46.6 |
| Son or daughter | 40 | 32.3 | 25 | 34.2 |
| Brother or sister | 18 | 14.5 | 12 | 10.8 |
| Paid employee | 52 | 41.9 | 34 | 30.6 |
| Others (Father in law- mother in law, nephew / niece and teacher) | 36 | 26.42 | 18 | 24.7 |
| Mother faced problems taking care of child at home^$^ | 86 | 46.5 | 39 | 51.3 |
| Mother does not participate in a social program along with special child^$^ | 152 | 82.2 | 57 | 75.0 |
| Reasons of not participating in the social program *(%)*  (multiple response) |  |  |  |  |
| Child does not like it and creates social nuisance | 23 | 69.7 | 14 | 77.8 |
| Mother does not enjoy it | 2 | 6.1 | 1 | 5.6 |
| People bully or question about the child | 13 | 39.4 | 5 | 27.8 |
| People show negative attitude towards the child when the child is taken outside home^$^ | 92 | 48.9 | 17 | 47.2 |
| Mother faces problem when take child outside, n (%)^$^ | 108 | 57.4 | 46 | 59.7 |
| Types of problems that mother faced when take child outside (multiple response) |  |  |  |  |
| Child’s act embarrasses mother in the society | 23 | 21.9 | 11 | 23.9 |
| Child walks away unnoticed and mother needs to pay very close attention | 30 | 28.6 | 17 | 37.0 |
| People bully at the child | 13 | 12.4 | 3 | 6.5 |
| Child gets hyper and uncontrollable | 62 | 59.0 | 24 | 52.2 |
| Mother is embarrassed about her child | 5 | 4.8 | 2 | 4.3 |
| child cannot act usual | 3 | 2.9 | 3 | 6.5 |
|  |  |  |  |  |
| ***Support of mother for own physical health^$^*** |  |  |  |  |
| Reported physical illness in the last 6 month, n(%) | 130 | 69.1 | 42 | 54.5 |
| Reported received treatment in last 6 month^$^ | 102 | 78.4 | 40 | 51.9 |
| Types of provider visited for a physical illness in the last 6 month (multiple response) |  |  |  |  |
| Doctor’s chambers | 98 | 52.1 | 44 | 57.1 |
| Private hospital | 61 | 32.4 | 29 | 37.7 |
| Clinic | 26 | 13.8 | 6 | 7.8 |
| Govt. hospital | 26 | 13.8 | 10 | 13.0 |
| Pharmacy | 7 | 3.7 | 2 | 2.6 |
| Satisfied with the health services received from a healthcare providers^$^ |  |  |  |  |
| Unsatisfied | 9 | 4.3 | 1 | 1.3 |
| Satisfied to some extent | 42 | 22.5 | 19 | 25.0 |
| Satisfied | 137 | 73.3 | 56 | 73.7 |
| ***Support of mother for mental health^$^*** |  |  |  |  |
| Any negative event took place in the last 6 months that caused mental pressure or trauma | 99 | 53.2 | 45 | 58.4 |
| Types of negative event (n=99) (multiple response) |  |  |  |  |
| Tension about child’s health or future | 32 | 39.0 | 15 | 41.7 |
| Social/ legal conflicts in family | 20 | 24.4 | 9 | 25.0 |
| Problem in source of income | 10 | 12.2 | 3 | 8.3 |
| Death or sickness of a near one | 9 | 11.0 | 4 | 11.1 |
| Sickness of husband | 9 | 11.0 | 0 | 0.0 |
| Child behaved abnormal and uncontrollable | 9 | 11.0 | 5 | 13.9 |
| Mother was sick or exhausted | 6 | 7.3 | 5 | 13.9 |
| Welfare of other children | 3 | 3.7 | 3 | 8.3 |
| Steps taken to reduce mental pressure (n=99) (multiple response)^$^ |  |  |  |  |
| Did nothing | 46 | 47.9 | 16 | 38.1 |
| Avoiding situations that cause trauma | 20 | 20.8 | 7 | 16.7 |
| Sharing with others | 18 | 18.8 | 12 | 28.6 |
| Sought advice of a doctor or a psychiatrist | 17 | 17.7 | 9 | 21.4 |
|  |  |  |  |  |
| ***Mothers’ experience with health service for the child with ASD*** |  |  |  |  |
| Physical illness of the child in the last 6 month^$^ | 92 | 48.9 | 38 | 49.4 |
| Satisfaction with the providers |  |  |  |  |
| Unsatisfied | 9 | 4.8 | 5 | 6.5 |
| Satisfied to some extent | 38 | 20.3 | 16 | 20.8 |
| Satisfied | 140 | 74.9 | 56 | 72.7 |
| Mother had an unpleasant experience with provider^$^ |  |  |  |  |
| Nothing significant | 130 | 71.4 | 51 | 71.8 |
| Behavior of the provider | 21 | 11.5 | 8 | 11.3 |
| Long waiting for the doctor | 18 | 9.9 | 8 | 11.3 |
| Facility environment was not conducive for the child | 7 | 3.8 | 2 | 2.8 |
| Child became irritated | 12 | 6.6 | 2 | 2.8 |
| Mother received any training on taking care of child^$^ | 122 | 64.9 | 56 | 72.7 |

^$^ Information of 4 participants was missing; SD: Standard Deviation

**Supplementary Table 2.** Feasibility of the psychosocial intervention program at the school settings

| *Variables* | Total=60 | % |
| --- | --- | --- |
| ***Acceptability*** |  |  |
| Satisfaction about the service from the counselors/providers |  |  |
| Very satisfied | 8 | 13.3 |
| Satisfied | 49 | 81.7 |
| Neutral | 3 | 5.0 |
| Level of satisfaction in the components of mental health services |  |  |
| *Counseling sessions (n=53)* |  |  |
| Positive attitude | 47 | 88.7 |
| Neutral attitude | 6 | 11.3 |
| *Meditation (n=31)* |  |  |
| Positive attitude | 24 | 77.4 |
| Neutral attitude | 7 | 22.6 |
| *Workshops (n=31)* |  |  |
| Positive attitude | 29 | 93.5 |
| Neutral attitude | 2 | 6.5 |
| *Psychiatrist visit (n=6)* |  |  |
| Positive attitude | 4 | 66.7 |
| Neutral attitude | 2 | 33.3 |
| Opportunities that facilitated mothers to avail the PCS services (multiple options) |  |  |
| Choice of integrating in to school was convenient (n=60) | 33 | 55.0 |
| Cost was an issue (n=60) | 18 | 30.0 |
| Supported having peace of mind (n=60) | 12 | 20.0 |
| Gave mothers space to get support for child care (n=60) | 11 | 18.3 |
| The counseling service fulfill the expectations of mothers | 54 | 90.0 |
| Reasons that mental health services meet the expectations- (multiple options) |  |  |
| Improvement in mental health condition (n=54) | 35 | 63.6 |
| Self empowerment with knowledge and good health(n=54) | 17 | 30.9 |
| To learn better about child care (n=54) | 8 | 14.5 |
| To have an opportunity to share feelings (n=54) | 4 | 7.3 |
| ***Benefit*** |  |  |
| Has there been any positive change in your daily life after taking the school counseling? | 53 | 88.3 |
| Types of improvement (multiple options) |  |  |
| Increased caring for myself (n=53) | 42 | 79.2 |
| Increased self-confidence (n=53) | 43 | 81.1 |
| Interest in daily activities has increased (n=53) | 41 | 77.4 |
| Reduced depression and stress (n=53) | 5 | 9.4 |
| The counseling service was helpful for the child care | 52 | 86.7 |
| Types of support if the service is helpful |  |  |
| Taking more care of the child | 27 | 51.9 |
| Stopped negative attitude /feeling about the child | 11 | 21.2 |
| Increased patience or acceptance about the child | 10 | 19.2 |
| Better communication with child | 3 | 5.8 |
| Mothers have done something new in last six months that gave you mental peace | 45 | 75.0 |
| Activities that improved the mental peace of mothers (n=45)  (multiple options) |  |  |
| Recreational activities | 22 | 48.9 |
| Follow religious activities | 14 | 31.1 |
| Positive changes in life | 12 | 26.7 |
| Did something new | 9 | 20.0 |
| Improved relationship | 5 | 11.1 |
| Skill building | 2 | 4.4 |
|  |  |  |
| ***Demand*** |  |  |
| Interested to receive this counseling facility in future | 58 | 96.7 |
| Recommendation for adopting additional measures to make this mental health services more effectives | 44 | 73.3 |
| Types of additional measures (multiple options) |  |  |
| Offer the service beyond the school hour (n=44) | 19 | 43.2 |
| Included other services or therapy (n=44) | 7 | 15.9 |
| Integrate the service in routine program (n=44) | 5 | 11.4 |
| Strengthen training modality by adding more means(n=44) | 5 | 11.4 |
| Involve family members and including child with ASD (n=44) | 3 | 6.8 |
| ***Barrier*** |  |  |
| Mother did not face any difficulties for availing the mental health services | 52 | 86.7 |
| Challenges/barriers that a mother may face while taking services from school (multiple options) |  |  |
| Cannot think of any (n=60) | 34 | 56.7 |
| Problems related to family or lack of family support (n=60) | 13 | 21.7 |
| No time due to other priorities (n=60) | 9 | 15.0 |
| Hesitation to avail care due to social stigma about autism (n=60) | 7 | 11.7 |
| Lack of financial support to avail care (n=60) | 1 | 1.7 |

**Supplementary Table 3.** Out of pocket cost of attending MENTHOL services borne by the mothers of children with ASD at schools (N=81)

| **Cost items** | **No. of participant** | **Number of events** | **Total Cost (BDT)*** | **Cost per mother (BDT)** | **Cost per session BDT)** |
| --- | --- | --- | --- | --- | --- |
| Transportation for attending session or a psychiatrist session at the school | 21 | 72 | 10,555 | 502 | 147 |
| Psychiatrist consultation at hospital | 0 | 0 | 0 | 0 | 0 |
| Medicine | 4 | 4 | 500 | 125 | 125 |
| Social Activities^*^ | 6 | 10 | 14500 | 2417 | 1450 |
| Total Cost in BDT | 81 | 318 | 25275 | 312 | 80 |
| Cost in USD | 81 | 318 | 303.06 | 3.74 | 0.96 |

- ^Those mothers who participatnd in the social program and paid^

**Supplementary Table 4.** Impact of Psychological Counseling Services on the change of depression

| DMS score | Pre intervention Mean±SD | Post intervention Mean±SD | Mean difference  (Relative change) | P-value^**^ (Within group) | Diff-in-Diff (between group)  (Relative change) | P-value^#^ interaction (Time × Group)^*^ |
| --- | --- | --- | --- | --- | --- | --- |
| Overall | 79.5 (±23) | 60.0(±20) | -19.5 (32%) | 0.004 |  |  |
| MDE | 97.8 (±12.1) | 69.9(±22.1) | -27.9 (40%) | P<0.001 | 11.3 (20%) | P<0.001 |
| Without MDE (n=59) | 72.7(±22.6) | 56.1(±18.1) | -16.6 (23%) | 0.003 |  |  |

^**^ Paired sample t-test for mean difference; ^#^ P-value of mean changes of DMS score in MDE compare to without MDE group over six months of intervention from baseline (general linear model).

**Supplementary Table 5.** Linear regression analysis between quality of life measured by EQ5D index and VAS score with the depression measured by DMS score and maternal factors.

|  | Difference of EQ5D index score | | Difference of EQ5D VAS score | |
| --- | --- | --- | --- | --- |
|  | Unadjusted beta coefficient  (95% CI)^*^ | P-value | Unadjusted beta coefficient (95% CI)^*^ | P-value |
| Difference DMS score | -0.003 (-0.006,0.001) | 0.102 | -0.213 (-0.370,-0.056) | 0.008^*^ |
| Demography information |  |  |  |  |
| Mother age | -0.001 (-0.009,0.008) | 0.839 | -0.247 (-0.682, 0.187) | 0.261 |
| Child age | 0.005 (-0.008,0.017) | 0.443 | -0.205 (-0.860, 0.451) | 0.536 |
| Housewife mothers (ref: working mothers) | 0.016 (-0.125,0.158) | 0.819 | -0.626 (-8.90, 7.65) | 0.881 |
| Living in nuclear family | 0.043 (-0.118,0.203) | 0.597 | 0.190 (-9.46,9.24) | 0.981 |
| Support system |  |  |  |  |
| Mother get support at home for take care of child | 0.011 (-0.117,0.138) | 0.869 | 3.42 (-2.52,9.38) | 0.255 |
| Mother who do not face difficulties for child care at home | 0.122 (0.044,0.239) | 0.043^*^ | 6.27 (0.684,11.85) | 0.028^*^ |
| Those mothers did not experience negative attitude for child | 0.009 (-0.110,0.129) | 0.877 | -0.792 (-6.48,4.90) | 0.782 |
| Those mother take their child at social program | -0.103 (-0.242,0.035) | 0.141 | -1.36 (-8.03,5.32) | 0.687 |
| Those mother who did not face difficulties outside with child | 0.151 (0.034,0.268) | 0.012^*^ | -0.53 (-7.67,6.61) | 0.883 |
| Seeking care for physical and mental health |  |  |  |  |
| Mother seeking care for physical illness | -0.016 (-0.136,0.105) | 0.797 | -0.539 (-7.55,6.47) | 0.879, |
| Mother attend workshop for child care | 0.012 (-0.124,0.147) | 0.864 | 3.67 (-4.15,11.49) | 0.353 |
| Mother attended mental health related workshop | 0.40 (-0.072,0.152) | 0.477 | 1.28 (-5.25,7.82) | 0.697 |

* All the analysis were controlled by the pre EQ5D index, pre EQ5D vas score and pre DMS score

**Supplementary Table 6.** Comparison between intervention and non-intervention mothers following the intervention period

| Factors | Intervention mothers | | non- intervention mothers | | P-value^#^ |
| --- | --- | --- | --- | --- | --- |
|  | N=60* | % | N=100 | % |  |
| *Care offered to the child with ASD* |  |  |  |  |  |
| Mothers faced any difficulty taking care of your child at home | 26 | 44.1 | 35 | 41.2 | 0.730 |
| Anyone in the family showed a negative attitude because of your child | 24 | 40.7 | 29 | 34.1 | 0.422 |
| Anyone in the family who took care of the child when the mother busy at home or outside home | 41 | 69.5 | 58 | 68.2 | 0.873 |
| Anyone in the family (other than husband) carried any responsibility to support the mother taking care of the child | 40 | 67.8 | 64 | 76.2 | 0.267 |
| Husband carried any responsibility to support the mother at home or outside home | 44 | 81.5 | 68 | 86.1 | 0.457 |
| Mother faced any difficulty with the child when took him outside home | 38 | 64.4 | 56 | 65.9 | 0.855 |
| Mother observed any positive change in the child in the past 6 months. | 52 | 88.1 | 63 | 74.1 | 0.039* |
| Types of improvement observed in the child in the past 6 months |  |  |  |  |  |
| Improvement of behavioral problems | 30 | 57.7 | 42 | 66.7 | 0.322 |
| Decreased dependency in daily activities | 33 | 63.5 | 46 | 73.0 | 0.271 |
| Increased communication skills | 43 | 82.7 | 48 | 76.2 | 0.393 |
| Increased vocational skills | 26 | 50.0 | 28 | 44.4 | 0.552 |
| Mother spent more time with the child | 29 | 49.2 | 15 | 17.6 | P<0.001** |
| *Social interactions* |  |  |  |  |  |
| Took child to a social events in the past 6 months | 53 | 89.8 | 75 | 88.2 | 0.765 |
| Number of times parents outing with children in the past 6 months^$^ | 70 (±85) |  | 68 (±90) |  | 0.761 |
| *Physical and mental health care* |  |  |  |  |  |
| Mother suffered from an illness in the past 6 months. | 42 | 71.2 | 49 | 57.6 | 0.098 |
| Mother sought treatment for illness in the past 6 months (intervention, n=42 and non-intervnetion, n=49) | 29 | 69.0 | 38 | 77.6 | 0.359 |
| Something happened in mother’s life that caused you mental pressure in the past 6 months? | 35 | 59.3 | 37 | 43.5 | 0.062 |
|  |  |  |  |  |  |
| What did you do in the past 6 months to reduce or remove your mental pressure? | n=23 |  | n=17 |  |  |
| Self control^b^ | 4 | 17.4 | 7 | 33.3 | 0.223 |
| Talked to others^b^ | 9 | 39.1 | 10 | 47.6 | 0.570 |
| Prayer^b^ | 6 | 26.1 | 10 | 47.6 | 0.138 |
| Consulted a psychologist | 2 | 8.7 | 0 | 0.0 | 0.167 |
| Took medicine/treatment/therapy | 5 | 21.7 | 2 | 10.0 | 0.298 |
| Consulted a doctor and got myself examined | 1 | 4.3 | 3 | 14.3 | 0.252 |
| Did nothing (intervention, n=36 and non-intervnetion, n=43) | 13 | 36.1 | 16 | 37.2 | 0.436 |
| Did you take any treatment/care for mental health? | 27 | 77.1 | 2 | 5.4 | P<0.001** |
|  |  |  |  |  |  |
|  |  |  |  |  |  |
|  |  |  |  |  |  |
| Any physical or mental health related issues hampered mother taking care of your child with ASD in the past 6 months? | 27 | 45.8 | 23 | 27.1 | 0.020** |
| Did you participate in training program related to child care for ASD in the past 6 months? | 27 | 45.8 | 19 | 22.4 | 0.010** |
| Did you attend any workshop on mental health in the past 6 months? | 42 | 71.2 | 16 | 18.8 | P<0.001** |
| Did you do anything in the last 6 months that gave you mental peace? | 44 | 74.6 | 42 | 49.4 | 0.002^*^ |
| *PHQ-9scores* |  |  |  |  |  |
| Minimal depression | 47 | 78.3 | 82 | 82.0 | 0.841 |
| Mild depression | 9 | 15.0 | 12 | 12.0 |  |
| Moderate depression | 4 | 6.7 | 6 | 6.0 |  |
| Proportions of mothers with depression (MDE) |  |  |  |  |  |
| Baseline MDE, n (%) | 15 | 62.5 | 2 | 6.9 | P<0.001 |
| End line MDE, n (%) | 4 | 33.3 | 1 | 5.8 | 0.054 |
| Reduction of % of mothers with MDE from baseline to end line, n (%) | 29.2 | | 1.1 | | P<0.001 |
| *Quality of life* | | | | | |
| EQ5D index, Baseline, Mean ±SD | 0.77±0.22 | | 0.76±0.23 | | 0.423 |
| EQ5D index, End line, Mean ±SD | 0.84±0.15 | | 0.85±0.16 | | 0.203 |
| EQ5D index, difference (baseline-endline) | -0.07 | | -0.09 | | 0.240^$^ |
| EQ5D VAS score, Baseline, Mean ±SD | 67.2±19.2 | | 70.1±16.2 | | 0.138 |
| EQ5D VAS score, Endline, Mean ±SD | 72.8±16.4 | | 73.9±18.3 | | 0.539 |
| EQ5D VAS score, difference (baseline-end line) | -5.8 | | -3.8 | | **0.021^$^** |

**^*^** 60 out of 81 intervention mothers participated in the end line survey. Twenty one mothers did not participate and the closing of school due to Ramadan(month of sacrifice and prayer for Muslims) during endline survey was the main reasons of loss to follow up;^$^ Outing with family member in restaurant, park, open playground, ^b^ The denominator of intervention mother was 23 and non-intervention mother was 27 **^$^**P-value indicates the difference of EQ5D score between the mothers service received and service not received group

# Chi-square test has applied until otherwise mentioned **;** Statistical significance at P<0.05^*^ and P<0.001**

**Supplementary file: Figure 3
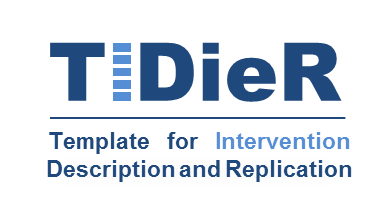
:The TIDieR(Template for Intervention Description and Replication) Checklist*:**

Information to include when describing an intervention and the location of the information

| **Item number** | **Item** | **Where located **** | |
| --- | --- | --- | --- |
|  |  | Primary paper  (page or appendix  number) | Other ^†^ (details) |
|  | **BRIEF NAME** |  |  |
| **1.** | Provide the name or a phrase that describes the intervention. | Psychological counseling services | **Study protocol:** Naheed, A., Koly, K. N., Ahmed, H. U., Akhter, S., Uddin, M. J., Fawzi, M. C. S., ... & Munir, K. (2017). Implementing a mental health care program and home-based training for mothers of children with autism spectrum disorder in an urban population in Bangladesh: Protocol for a feasibility assessment study. *JMIR research protocols*, *6*(12), e8260. |
|  | **WHY** |  |  |
| **2.** | Describe any rationale, theory, or goal of the elements essential to the intervention. | The psychological counseling service has an important role in lowering depression and improving health-related quality of life. The intervention is more beneficial in mothers who are experiencing major depressive episode. |  |
|  | **WHAT** |  |  |
| **3.** | Materials: Describe any physical or informational materials used in the intervention, including those provided to participants or used in intervention delivery or in training of intervention providers. Provide information on where the materials can be accessed (e.g. online appendix, URL). | At each school, a counseling center was established, with one qualified female psychologist providing counseling to intervention mothers. A counseling module was created with the assistance of an Expert Working Group (EWG) in Bangladesh comprised of psychiatrists, psychologists, and other relevant professionals. The intervention includes psychoeducation, an assessment of the mothers' strengths and weaknesses, and the sharing of the management plan with the mothers,  psychological reconstruction, behavior therapy-graded activity, and developing a mother's community and involving mothers in community-related activities | _____________ |
| **4.** | Procedures: Describe each of the procedures, activities, and/or processes used in the intervention, including any enabling or support activities. | On the basis of a mother's need, a psychologist determined the number of sessions and the intervals between them. A depression measurement scale was used by the psychologist before each session to check the mental health of the respondents. Every month, a psychiatrist from the National Institute of Mental Health (NIMH),Bangladesh visited each school and evaluated the records kept by the psychologist, as well as offer essential recommendations on the need for any proper care for the mothers who have been diagnosed with a major depressive episode.  Following a face-to-face meeting, the psychiatrist appraised the extra requirement of a mother with MDE if recommended by the record review or clinically diagnosed by the psychologist. | _____________ |
|  | **WHO PROVIDED** |  |  |
| **5.** | For each category of intervention provider (e.g. psychologist, nursing assistant), describe their expertise, background and any specific training given. | Female psychologist | _____________ |
|  | **HOW** | The intervention provided by Face to face mode between psychologist and participants. |  |
| **6.** | Describe the modes of delivery (e.g. face-to-face or by some other mechanism, such as internet or telephone) of the intervention and whether it was provided individually or in a group. |  | _____________ |
|  | **WHERE** |  |  |
| **7.** | Describe the type(s) of location(s) where the intervention occurred, including any necessary infrastructure or relevant features. | In the special school for children with autism | _____________ |
|  | **WHEN and HOW MUCH** |  |  |
| **8.** | Describe the number of times the intervention was delivered and over what period of time including the number of sessions, their schedule, and their duration, intensity or dose. | Any mother who stepped in with or without a prior history of depression or other mental health disorders received the intervention during school hours for over six months. Every two months, the psychologists gave workshops in each school, with group meditation sessions after each program. | _____________ |
|  | **TAILORING** |  |  |
| **9.** | If the intervention was planned to be personalised, titrated or adapted, then describe what, why, when, and how. | All mothers who were referred to the NIMH,B for advanced mental health treatment were entitled for free antidepressant, anxiolytic, and antipsychotic drugs if clinically treated. | _____________ |
|  | **MODIFICATIONS** |  |  |
| **10.^ǂ^** | If the intervention was modified during the course of the study, describe the changes (what, why, when, and how). | Not relevant for this study. | _____________ |
|  | **HOW WELL** |  |  |
| **11.** | Planned: If intervention adherence or fidelity was assessed, describe how and by whom, and if any strategies were used to maintain or improve fidelity, describe them. | The mothers scheduled 73 sessions in the first month of the intervention period, and 60 sessions were held (85% ). In the last month of the intervention, 53 sessions were scheduled and 52 were held (98% ). Seventy-five percent of mothers took part in the exit interview, and over 95 percent said they were satisfied with the intervention. | _____________ |
| **12.^ǂ^** | Actual: If intervention adherence or fidelity was assessed, describe the extent to which the intervention was delivered as planned. | _____________ | _____________ |

** **Authors** - use N/A if an item is not applicable for the intervention being described. **Reviewers** – use ‘?’ if information about the element is not reported/not sufficiently reported.

† If the information is not provided in the primary paper, give details of where this information is available. This may include locations such as a published protocol or other published papers (provide citation details) or a website (provide the URL).

ǂ If completing the TIDieR checklist for a protocol, these items are not relevant to the protocol and cannot be described until the study is complete.

* We strongly recommend using this checklist in conjunction with the TIDieR guide (see *BMJ* 2014;348:g1687) which contains an explanation and elaboration for each item.

* The focus of TIDieR is on reporting details of the intervention elements (and where relevant, comparison elements) of a study. Other elements and methodological features of studies are covered by other reporting statements and checklists and have not been duplicated as part of the TIDieR checklist. When a **randomised trial** is being reported, the TIDieR checklist should be used in conjunction with the CONSORT statement (see [www.consort-statement.org](http://www.consort-statement.org)) as an extension of **Item 5 of the CONSORT 2010 Statement.** When a **clinical trial** **protocol** is being reported, the TIDieR checklist should be used in conjunction with the SPIRIT statement as an extension of **Item 11 of the SPIRIT 2013 Statement** (see [www.spirit-statement.org](http://www.spirit-statement.org)). For alternate study designs, TIDieR can be used in conjunction with the appropriate checklist for that study design (see [www.equator-network.org](http://www.equator-network.org)).
